# Coin Paradox Spin–Orbit Interaction Enhances Magneto-Optical Effect and Its Application in On-Chip Integrated Optical Isolator

**DOI:** 10.1186/s11671-021-03634-8

**Published:** 2021-12-07

**Authors:** Hao Hu, Jiwei Qi, Qiang Wu, Xianhui Fu, Hongjin Wu, Sihao Zhang, Zongqiang Chen, Jing Chen, Jianghong Yao, Xuanyi Yu, Qian Sun, Jingjun Xu

**Affiliations:** 1 Key Laboratory of Weak-Light Nonlinear Photonics, Ministry of Education, TEDA Institute of Applied Physics and School of Physics, Nankai University, Tianjin, 300457 China; 2 Collaborative Innovation Center of Extreme Optics, Shanxi University, Taiyuan, 030006 Shanxi China

**Keywords:** Coin paradox, Spin–orbit interactions, Magneto-optic effect, Isolator

## Abstract

We designed a simple on-chip integrated optical isolator made up of a metal–insulator–metal waveguide and a disc cavity filled with magneto-optical material to enhance the transverse magneto-optical effect through the coin paradox spin–orbit interaction (SOI). The simulation results of the non-reciprocal transmission properties of this optical structure show that a high-performance on-chip integrated optical isolator is obtained. The maximum isolation ratio is greater than 60 dB with a corresponding insertion loss of about 2 dB. The great performance of the optical isolator is attributed to the strong transverse magneto-optical effect, which is enhanced by the coin paradox SOI. Moreover, the enhancement of the transverse magneto-optical effect through the coin paradox SOI is more substantial for smaller azimuthal mode number *n*. Benefiting from this, the transverse magneto-optical effect remains strong in a wide wavelength range. Additionally, a smaller cavity has a stronger transverse magneto-optical effect in the same wavelength range. Our research provides a new perspective for creating highly integrated magneto-optical devices.

## Introduction

Optical isolators based on non-reciprocal transmission are the key photonic elements in optical telecommunications and optical information. To achieve a more integrated optical isolator, many methods, such as the use of magneto-optical effect [1–5], topology [6], nonlinear effects [7–12] and parity-time symmetry breaking [13–15], have been developed. Among these, the magneto-optical effect is still a hotspot. So far, however, the devices created have typically been in large scales [2, 16] because the magneto-optical effect is mostly weak in these cases.

Surface plasmon polariton (SPP) can break the diffraction limit [17, 18] and has excellent potential in integrated optics [19–21], especially after the improvement in the problem regarding high loss of SPP [22]. SPP has transverse spin angular momentum (TSAM) [23–25], which can induce a magneto-optical effect to realize a non-reciprocal transmission similar to the longitudinal spin angular momentum (LSAM) of light [26–28]. However, it is difficult to miniaturize the isolator based on the transverse magneto-optical effect of SPP due to the weak magneto-optical effect. There are two key reasons resulted in the weak transverse magneto-optical effect of SPP; one is the small magneto-optic coefficient of the magneto-optic materials, and the other is that the transverse spin of SPP is not circular, but elliptical [26]. At present, a variety of magneto-optical materials with large magneto-optic coefficients have been fabricated and applied to light isolators [4, 29–32]. This brings hope for creating miniaturized optical isolators with plasmonic structures. But, on the other hand, the elliptical transverse spin of SPP is still the bottleneck for the application of the transverse magneto-optical effect. The discovery of new methods to enhance the transverse magneto-optical effect is still desired.

Spin and orbital angular momenta (SAM and OAM) are two distinct light components. These can interact with each other effectively, that is, via spin–orbit interaction (SOI). Many essential and valuable optical effects based on SOI of light have been discovered, including spin Hall effect, quantum spin Hall effect, topology, etc. Coin paradox is a fascinating natural phenomenon, which shows a distinctive SOI, that circumferential orbit causes the rotation of the spin change. Thus, coin paradox SOI may be a new physical mechanism to regulate the transverse magneto-optical effect of SPP.

This work reports the design of a simple on-chip integrated optical isolator composed of a metal–insulator–metal (MIM) waveguide and a disc cavity filled with a magneto-optical material. In this optical isolator structure, the effective enhancement of the transverse magneto-optical effect was confirmed through coin paradox SOI. Benefiting from the enhanced transverse magneto-optical effect in the optical isolator structure, the forward and backward resonant valleys in the transmission spectra were wholly separated from each other when magneto-optical parameter $\varepsilon_{xy} \ge 0.04$. A high-performance on-chip integrated optical isolator was obtained, for which the maximum isolation ratio (IR) was greater than 60 dB and the corresponding insertion loss (IL) was about 2 dB. Due to the unique properties of coin paradox SOI in the optical isolator structure, the improvement of the transverse magneto-optical effect is more substantial for smaller azimuthal mode number n. The transverse magneto-optical effect remained strong in a wide wavelength range. Furthermore, a greater transverse magneto-optical effect appeared in a smaller disc cavity, which could effectively surmount the broadening of the resonance valleys induced by the smaller cavity. The strong transverse magneto-optical effect structure developed herein has huge application potential in on-chip highly integrated magneto-optical devices, optical isolators, magneto-optical switches, magnetic sensors, etc.

## Methods

**Fig. 1:**
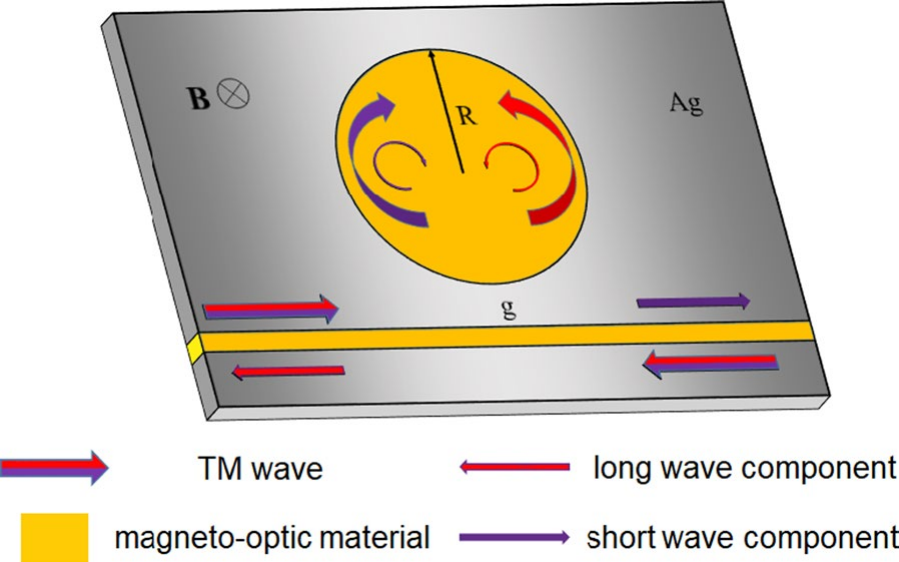
Schematic illustration of the optical isolator structure composed of a MIM waveguide and a disc cavity. The MIM waveguide and the disc cavity are filled with magneto-optical material and remain under a static magnetic field

Figure 1 shows the schematic illustration of the proposed optical isolator structure composed of a MIM waveguide and a disc cavity. The radius (*R*) of the disc cavity was set to 540 nm, the width of the MIM waveguide d was set to 50 nm and the gap between the disc cavity and the MIM waveguide g was set to 16.6 nm. The metal is silver, whose frequency-dependent complex relative permittivity is characterized by the Drude model:

1 $$\varepsilon_{m} (\omega ) = \varepsilon_{\infty } - \omega_{p}^{2} /\omega (\omega + i\gamma )$$

Here, $\varepsilon_{\infty }$ is the dielectric constant at an infinite frequency, *γ* is the electron collision frequency, $\omega_{p}$ is bulk plasma frequency, and *ω* is the angular frequency of the incident light. The parameters put into Eq. (1) were $\varepsilon_{\infty }$ = 3.7, $\omega_{p}$ = 9.1 eV, *γ* = 0.018 eV [33]. In order to excite the SPPs, the input light was set to transverse magnetic (TM) plane wave.

The disc cavity and the MIM waveguide were filled with magneto-optical material, and a transverse static magnetic field is applied. The effect of static magnetic field on magneto-optical materials was largely reflected in the dielectric tensor of materials. For anisotropic magneto-optical materials, a static magnetic field **B** can be applied along the z-direction, where the dielectric tensor can be expressed as:

2 $${{\varvec{\upvarepsilon}}} = \left( {\begin{array}{*{20}c} {\varepsilon_{xx} } & { - i\varepsilon_{xy} } & 0 \\ {i\varepsilon_{xy} } & {\varepsilon_{yy} } & 0 \\ 0 & 0 & {\varepsilon_{zz} } \\ \end{array} } \right)$$

The magneto-optical material is set to bismuth-doped yttrium iron garnet (Bi: YIG). Garnet belongs to the cubic crystal structure and is isotropic, so the diagonal elements of its dielectric tensor are identical, that is, $\varepsilon_{xx} = \varepsilon_{yy} = \varepsilon_{zz} = \varepsilon_{0} = n^{2}$. The dielectric constant $\varepsilon_{0}$ of the diagonal element is set to 4.84, the typical refractive index of YIG near the wavelength of 1.5 μm [34]. Recently, experiments have proved that the $\varepsilon_{xy}$ can be greater than 0.3 [35] and the theoretically predicted [36] $\varepsilon_{xy}$ is much greater than that obtained by experiment. In this work, the value of $\varepsilon_{xy}$ was set from 0 to 0.3. This device can be fabricated by metal-assisted chemical etching [37, 38] and electron beam lithography (EBL).

Commercial software COMSOL Multiphysics was employed for model building and simulation calculations based on the finite element method (FEM). For the convenience of research, the whole structure was two-dimensional. The passing Pointing vector **S** was integrated at the entrance and exit ends to obtain the entrance energy $P_{\text{in}}$ and the exit energy $P_{{{\text{out}}}}$, $P_{{{\text{in}}}} = \int {{\mathbf{S}}_{1} \bullet {\text{d}}{\varvec{s}}_{1} }$, $P_{{{\text{out}}}} = \int {{\mathbf{S}}_{2} \bullet {\text{d}}{\varvec{s}}_{2} }$ and the transmittance $T = 10{*}\lg \left( {P_{{{\text{out}}}} /P_{{{\text{in}}}} } \right)$ dB. IL is the backward transmission rate at the forward isolation wavelength and is calculated using the transmittance data obtained in the simulation. Light input was given from the left of the MIM waveguide, and its output from the right is labeled as ‘forward’ in this paper. Contrarily, the light input from the right of the MIM waveguide resulted in the output from the left, and called ‘backward’.

## Results and Discussion

As shown in Fig. 1, the disc cavity supports a fascinating coin paradox SOI. For example, for the mode TM_(0,*n*)_, the transverse spin and the orbit rotation of SPP lie in the same direction. SPP travels around the disc cavity one turn, and the electric field vector rotates *n* + 1 turns. The circular orbit causes the extra turn. This effect is similar to the coin paradox and forms a unique SOI. The coin paradox SOI is more significant for smaller n. The simulation results confirm that the coin paradox SOI can enhance the transverse magneto-optical effect effectively.

**Fig. 2:**
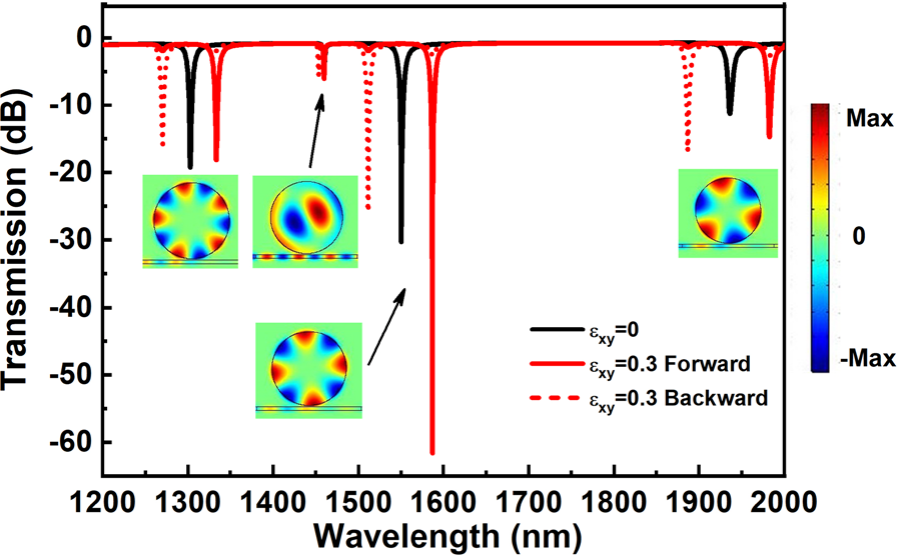
The total transmission spectra of the optical isolator structures for different $\varepsilon_{xy}$. The solid black line shows the transmission spectrum for $\varepsilon_{xy} = 0$, solid red line for $\varepsilon_{xy} = 0.3$ forward and the dotted red line for $\varepsilon_{xy} = 0.3$ backward. The insets below the transmission spectra are the intensity distributions of the z component of the magnetic field, corresponding to the case of $\varepsilon_{xy} = 0$

Figure 2 shows the transmission spectra of the optical isolator structures for different $\varepsilon_{xy}$. For the case of $\varepsilon_{xy} = 0$, forward and backward transmission spectra overlap, the transmission spectrum is shown as solid black line. The solid red line shows the transmission spectrum for the case of $\varepsilon_{xy} = 0.3$ forward, dotted red line for the case of $\varepsilon_{xy} = 0.3$ backward. As shown in Fig. 2, there are four prominent transmission valleys in each transmission spectrum. For the case of $\varepsilon_{xy} = 0$, four transmission valleys are located at 1936.0 nm, 1550.2 nm, 1460.0 nm and 1302.5 nm, respectively. For two-dimensional finite-element modeling, the resonances of the disc cavity are characterized by two integers (m_i_, n_i_) that count the radial and azimuthal antinode. According to the intensity distributions of the z component of the magnetic field shown in the insets, the four transmission valleys induced by resonant modes are: TM_0,3_, TM_0,4_, TM_1,1_ and TM_0,5_. In this paper, we focused mainly on the transverse magneto-optical effect of SPP, and so the resonant modes: TM_0,3_, TM_0,4_ and TM_0,5_ were researched in detail.

**Fig. 3:**
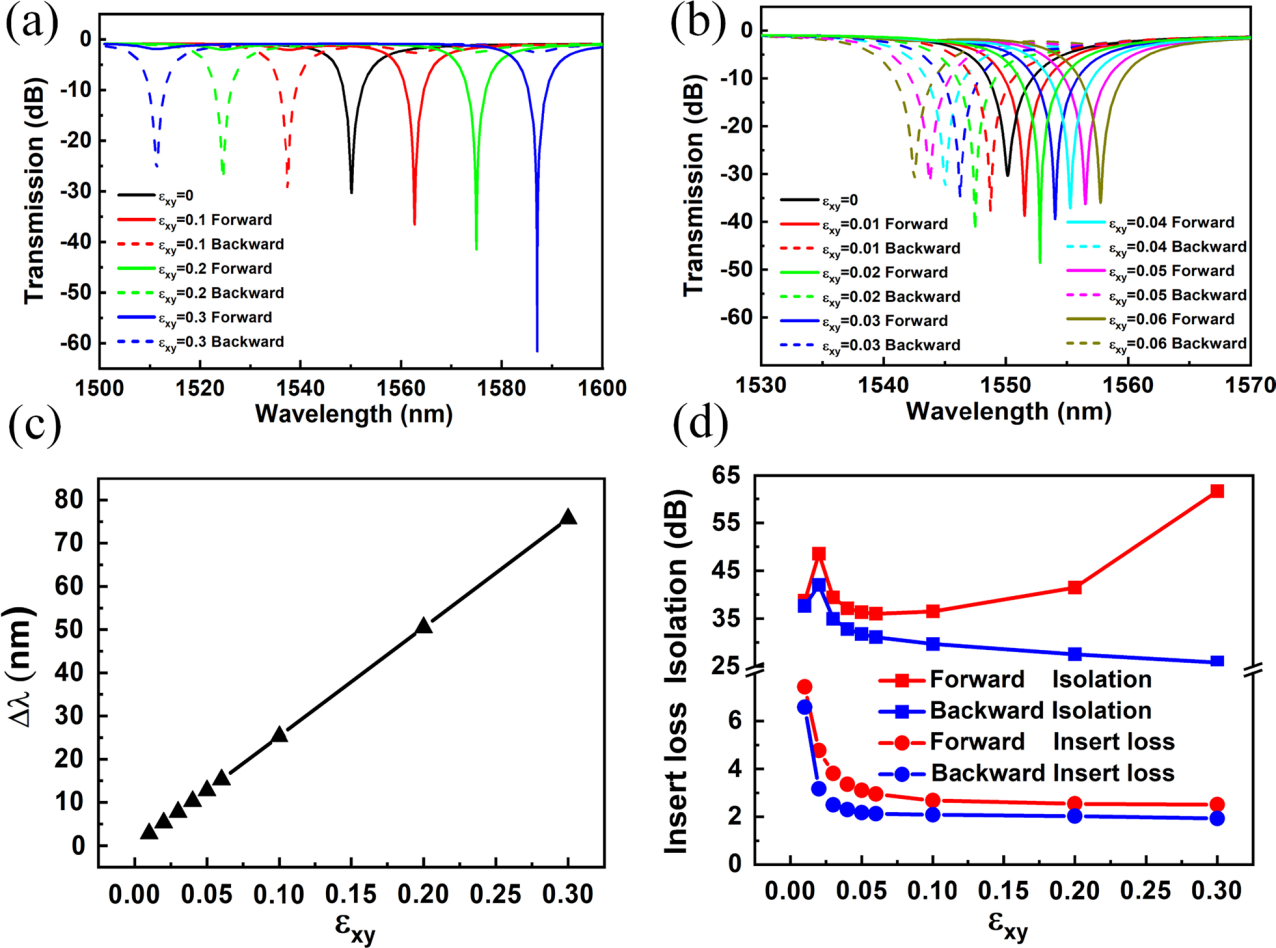
The transmission spectra, wavelength splitting, IR and IL in mode TM_0,4_. **a**, **b** The transmission spectra of light from different propagation directions coupled into the disc cavity having different $\varepsilon_{xy}$. **c**, **d** The linear graphs of wavelength splitting, IR, and IL as a function of $\varepsilon_{xy}$

Initially, the isolation performance of the optical isolator structure of resonant mode TM_0,4_ was studied. Figure 3a, b show the transmission spectra of the optical isolator structures of the resonant mode TM_0,4_ with different $\varepsilon_{xy}$. Without any magnetic field, the transmission valley is located at about 1550.2 nm. On applying the magnetic field, the transmitted valley has a red-shift as the SPP traveled forward and a blue-shift almost symmetrically as the SPP traveled backward. Thus, the splitting of forward and backward resonant valleys was observed. With the increase in the value of magneto-optical parameter $\varepsilon_{xy}$, the wavelength shifted and the splitting increased. Figure 3c shows the curve of the splitting of forward and backward resonant valleys varying with the magneto-optical parameter $\varepsilon_{xy}$. As shown in Fig. 3c, the splitting is practically positively related to the magneto-optical parameter $\varepsilon_{xy}$. Figure 3d displays the IR and the IL of the optical isolator structure of resonant mode TM_0,4_ for different $\varepsilon_{xy}$. With the increase in the value of $\varepsilon_{xy}$, both the forward and backward IL decreased. Besides, when $\varepsilon_{xy} \ge 0.05$, the IL was as small as about 2 dB and remained stable. This means that the forward and backward resonant valleys were entirely separated from each other. The forward and backward IR exhibited different change curves as the $\varepsilon_{xy}$ increased. As shown in Fig. 3d, we get the maximum IR greater than 60 dB with a corresponding IL of about 2 dB. The IR was determined by the depth of the transmission valley. It depends on the coupling distance between the MIM waveguide and the disc cavity. So, the IR can be fine-tuned by changing the gap between the MIM waveguide and the disc cavity, *g*. The relevant results show that the large magneto-optical effect exists in the optical isolator structure presented herein, and as a result, a high-performance on-chip integrated optical isolator is obtained.

**Fig. 4:**
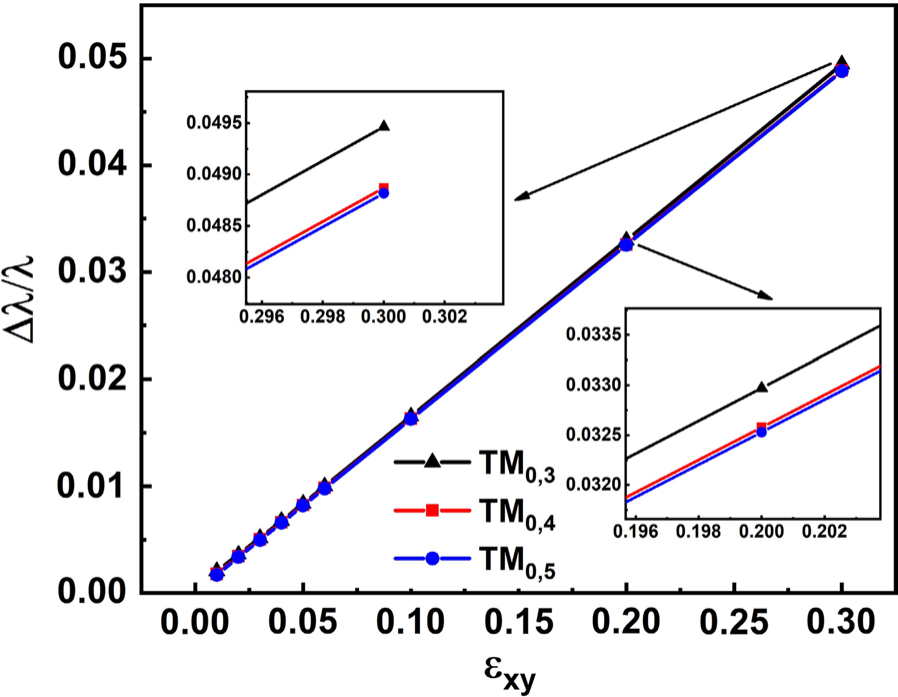
Line graph of the $\Delta \lambda /\lambda$ ratio varying with $\varepsilon_{xy}$ for different modes. The insets are the partially enlarged view of data points when $\varepsilon_{xy} = 0.2$ and $\varepsilon_{xy} = 0.3$

The enhancement of transverse magneto-optical effect by the coin paradox SOI will be more significant for smaller azimuthal mode number n. The simulation results can be used to prove this law. As shown in Fig. 2, for the cases of TM_0,5_, TM_0,4_ and TM_0,3_, the splitting $\Delta \lambda$ increased with the decrease in the azimuthal mode number n. To accurately compare the intensity of the transverse magneto-optical effect of different modes, line graphs of the $\Delta \lambda /\lambda$ ratio varying with $\varepsilon_{xy}$ for different modes are plotted in Fig. 4. As shown in Fig. 4, for three different modes, $\Delta \lambda /\lambda$ ratio just has a slight change. Besides, as shown in the insets, $\Delta \lambda /\lambda$ ratios of TM_0,5_ and TM_0,4_ are almost the same and that of TM_0,3_ is the largest. These simulation results are contrary to the theory reported in Ref. [26]. For the cases of TM_0,5_, TM_0,4_ and TM_0,3_, the resonance wavelength increased with the decrease in the azimuthal mode number n, which is clearly shown in Fig. 2. As the wavelength increased, the absolute value of the metal's dielectric permittivity $\varepsilon_{M}$ increased rapidly, resulting in a decrease in $\beta_{SPP}$. According to the theory in Ref. [26], the transverse magneto-optical effect was expected to be weakened, and the $\Delta \lambda /\lambda$ ratio to be smaller. Therefore, the present simulation results are contrary to the theory in Ref. [26]. The enhancement in the transverse magneto-optical effect by the coin paradox SOI can solve this contradiction between the simulation results and the theory in Ref. [26]. As mentioned above, the coin paradox SOI is more significant for smaller azimuthal mode number n. Thus, the enhancement in the transverse magneto-optical effect by the coin paradox SOI can cancel or even overtakes the weakening triggered by the increase in wavelength. In addition, another conclusion can be drawn that the abnormally large transverse magneto-optical effect mentioned in this work is caused by the coin paradox SOI and remains strong in a large wavelength range.

**Fig. 5:**
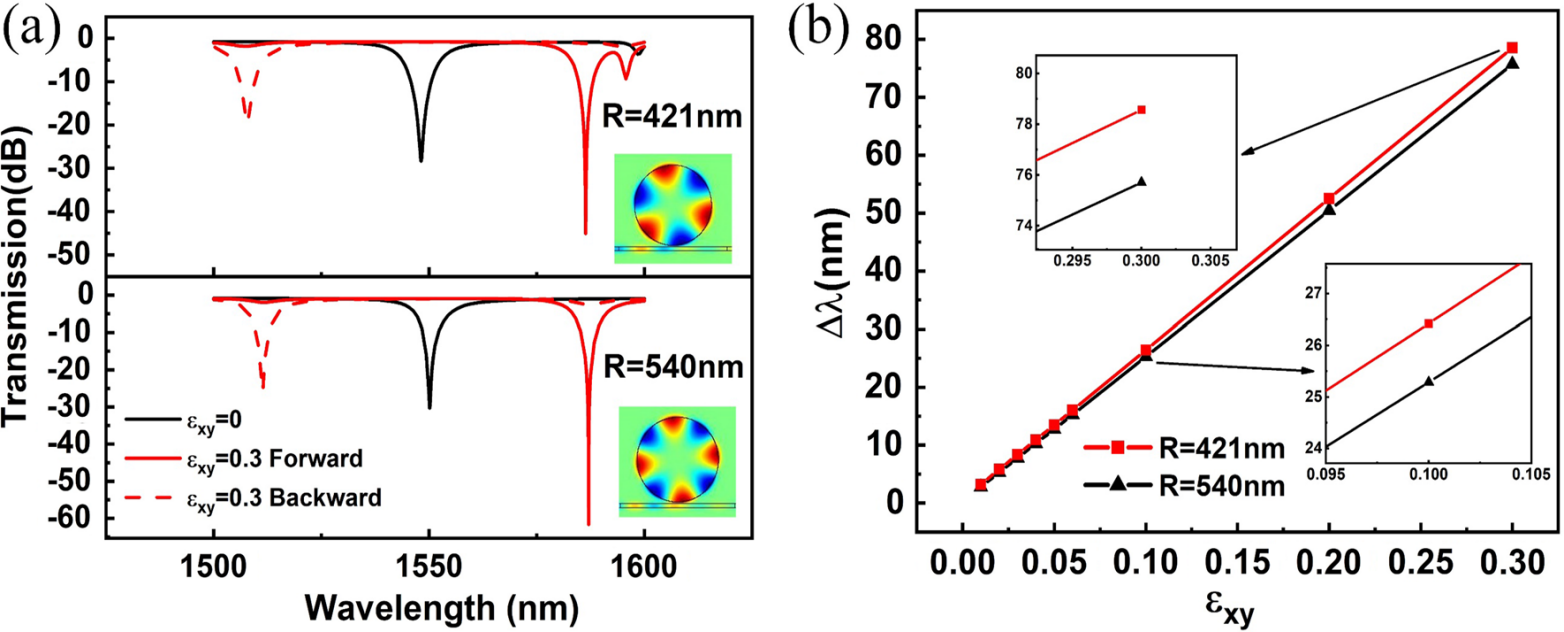
The transmission spectra and wavelength splitting of the disc cavity with different radii. **a** The transmission spectra of light from different propagation directions are coupled into a disc cavity with different radii. The insets correspond to the intensity distribution of the z component of the magnetic field when $\varepsilon_{xy} = 0$. **b** Linear graph of wavelength splitting for disc cavity with different radii

For smaller azimuthal mode number n, the enhancement of the transverse magneto-optical effect by the coin paradox SOI is more significant. Therefore, a smaller cavity will have a greater transverse magneto-optical effect in the same wavelength range, that is, a greater wavelength splitting. To confirm this conclusion, the radius of disc cavity *R* was set to a smaller value, 421 nm. The transmission spectrum of smaller cavity *R* = 421 nm is shown in Fig. 5a, and compared to that of bigger cavity *R* = 540 nm. It can be seen that TM_0,3_ for smaller cavity *R* = 421 nm and TM_0,4_ for bigger cavity *R* = 540 nm are both located at about 1550 nm. The linear graph of wavelength splitting changing with the $\varepsilon_{xy}$ for different radii of disc cavity is plotted in Fig. 5b. It is obvious that the wavelength splitting of the smaller cavity is larger than that of the bigger cavity, which agrees with our expectations. Moreover, the enhancement of the transverse magneto-optical effect by the coin paradox SOI is proven again.

It's well known that with the decrease in the radius of the disc cavity, the full width at half maxima (FWHM) of the spectral line will increase. Bigger FWHM has been the main insurmountable sticking point that hinders the application of cavities with smaller model volumes. The change in FWHM induced by the change of the $\varepsilon_{xy}$ can be ignored. With the radius of the disc cavity decreasing from 540 to 421 nm, the FWHM increased from about 9.914 nm to about 10.811 nm. With the decrease in the radius of the disc cavity, the FWHM increased by about 0.897 nm. This linear expansion can be effectively compensated by increased splitting. For instance, when $\varepsilon_{xy} = 0.1$, the increase in wavelength splitting was about 1.130 nm. When $\varepsilon_{xy} = 0.3$, the increase in wavelength splitting was about 2.850 nm, much greater than 0.897 nm. Therefore, the optical isolator structure presented here has a greater application potential at a smaller size and is more conducive to a higher degree of optical integration.

## Conclusion

In summary, a simple on-chip integrated optical isolator composed of a MIM waveguide and a disc cavity filled with magneto-optical material was designed. In this optical isolator structure, novel coin paradox spin–orbit interaction exists, enhancing the transverse magneto-optical effect effectively. Moreover, the enhancement is more significant for smaller azimuthal mode number n. Based on the enhanced transverse magneto-optical effect, a high-performance on-chip integrated optical isolator was obtained. The maximum IR was found to be greater than 60 dB with IL of about 2 dB. The transverse magneto-optical effect remains strong in a wide wavelength range. Furthermore, the greater transverse magneto-optical effect of the smaller cavities is verified, which can surmount the broadening of the resonance valleys induced by the smaller cavity effectively.

## Data Availability

All data supporting the conclusions of this article are included within the article.

## Abbreviations

SPP: Surface plasmon polariton
TSAM: Transverse spin angular momentum
LSAM: Longitudinal spin angular momentum
SAM: Spin angular momenta
OAM: Orbital angular momenta
SOI: Spin–orbit interaction
MIM: Metal–Insulator–Metal
IR: Isolation ratio
IL: Insertion loss
TM: Transverse magnetic
Bi: YIG: Bismuth-doped Yttrium Iron Garnet
FEM: Finite element method
FWHM: Full width at half maxima
EBL: Electron beam lithography
